# A Treatise on the Forces Which Produce the Organization of Plants. With an Appendix, Containing Several Memoirs on Capillary Attraction, Electricity, and the Chemical Action of Light

**Published:** 1845-07

**Authors:** 


### ART. XIV.

*A Treatise on the Forces which produce the Organization of Plants. With an Appendix, containing several Memoirs on Capillary Attraction, Electricity, and the Chemical Action of Light.* By JOHN WILLIAM DRAPER, M.D. Professor of Chemistry in the University of New York. With Four Plates.—*New York,* 1844. 4to, pp. 324.

THE handsome volume before us is by far the most original contribution to physiology and its allied sciences, that we have ever received from the American press. Dr. Draper has long been known to scientific men on this side of the Atlantic, as a most industrious and sagacious explorer of that fertile province which lies between physics and physiology. The papers which he has published, from time to time, in our scientific journals, have given evidence of his skill as an experimenter, and of his acumen as a reasoner. We have rejoiced, therefore, to see that they seemed likely to aid in the unravelling of those mysteries, which the physiologist continually encounters in the prosecution of his researches into the dependence of life upon external conditions. And we were prepared to give a hearty welcome to the present treatise, which embodies the physiological inferences to which the author has been led by his experimental investigations.

Unlike too many who call themselves chemical philosophers, our author has thought it incumbent upon him to make himself acquainted with the present state of opinion upon physiological subjects, as taught in the best schools; and to take this as the groundwork of his arguments. Consequently we have nowhere encountered, in perusing his treatise, those hasty assumptions and sweeping generalizations, which disfigure the works of too many of our scientific reasoners. On the contrary, we have been struck by the clearness and accuracy of his views on physiological subjects; indicating, we think that he has not taken them upon trust from others, but has made them his own by independent observation. The style of the work is something more ornate than we are accustomed to expect in a grave philosophical treatise; but we do not know that it is the worse for this, as the clearness of its statements and the closeness of its arguments do not seem thereby impaired.

Owing to the many other topics which crowd upon our attention, we must content ourselves with a much less full account of this work than its merits deserve; and must confine ourselves to those departments of it which have an especial bearing upon animal physiology. From the introductory chapter, containing general remarks on the influence of physical agents on organization and life, we shall select a few disconnected passages, as specimens of our author's style and manner of treating the subject.

"In a philosophical point of view, it was the office of the 17th century to unfold the doctrine of universal gravitation, to assign proper causes for the motions of the celestial bodies, and to develope the great doctrines of astronomy. It was the office of the 18th to lay the foundations of physics and chemistry, or of that group of sciences which embraces the relations and reactions of atoms. It is the office of the 19th to discover the laws which obtain in the complicated structure of animated beings,—those laws which give rise to the mysterious phenomena which we call life." (p. 1.)

"In their origin, all those important ideas, which now constitute modern science, have been obscurely and imperfectly set forth. It is not given to the human mind, when it emerges from the darkness of ignorance, any more than to the human eye, when it emerges from physical darkness into sunshine, to see all objects which are before it, in their proper aspect and position. A period of time must elapse, during which we become accustomed to the light." (p. 2.)

"Each one of the various changes in the universe, no matter whether it concerns organic or inorganic nature, has been the result of the action of some determining cause. The countless systems of phenomena, which have arisen, are all connected together as systems of effects. In a web, as it passes from the loom, the different threads interlace with one another; and though we soon cease to identify each, as it pursues its sinuous way, we know that the last is connected with the first: and in the web of nature, each event has been brought into relation with others that have gone before it, and others that have succeeded it, and all are intertwined together, as a series of causes and effects." (p. 4.)

"From the day when organization first commenced on the surface of the earth, the law which it has followed has been a law of progress and of evolvement. A myriad types of life have been created, and myriads of living forms produced; and the last is the highest. Even with us the same thing is going on; advances in knowledge are advances in power. The civilized man of these days is a wholly different being from the man who lived a thousand years ago; and the conditions which determine his position have totally changed. With us, the position both of empires and of individuals is fixed by the possession of knowledge,—knowledge which is incessantly on the advance. Wherever intelligence has been given, there is a requirement to join in the advancing march. The Indian stands still, and the penalty is death." (p. 8.)

"From these considerations, therefore, we may gather that the laws of nature contain provisions for the extinction and removal of successive races; operations which are carried on by the action of physical powers. As the death of an individual arises from the action of external agents, so, in the same manner, does the disappearance of a tribe: and hence we see that, as existence is under this control, it cannot take place except when physical circumstances conspire; as they change, so, also, must the various forms of life undergo corresponding mutations." (p. 8.)

"What, then, are the final impressions left upon our minds by these general considerations? They teach us that Life never occurs except in regions to which the imponderable agents can have access, an observation which is equally true of vegetable and of animal forms; that elementary organization directly or indirectly arises from the plastic energy of those all-pervading forces. Whether we consider the organic or inorganic world, all things around us are in incessant changes,—changes which result from the fixed operation of invariable laws; that, of the successive tribes of beings which have peopled our earth, each series may be regarded as expressing the general relation of all physical agents at the time of its existence, the brilliancy of the sun, the pressure of the air, and other such conditions; for we see that, between those conditions and the organization of the structures considered, there are fixed relations; that in the more highly complicated forms of being, mutations more readily take place, and in all, time enters as an element; that in the same way that whole races have disappeared from the face of the earth, and have become extinct, so also, do individuals die and atoms change; that whatever motion is accomplished, or whatever change is brought about, there is a consumption of material or an expenditure of force; that, as the surface of the earth is continually remodelled by physical agents, so are the vicissitudes through which organized forms pass determined by physical powers, and bring about physical ends. The passage of a comet, never more to return, in a hyperbolic orbit past the sun, is a result of the same general law that keeps a planet revolving in the repeated circles; the extinctions of races which have heretofore taken place, or which are going on before us, are not brought about by a direct intervention of supernumerary forces, but are the constant results of those which are always in action. If, moreover, our thoughts are directed to the relations which exist between climates and the character of races, the distribution of vegetables and animals; if we observe the antagonization of these great classes in the result of their vital processes, their position as respects the atmosphere, the control which astronomical events possess over everything, the action which currents in the air or currents in the sea exercise over the distribution of animated forms, and even over the well being of man, we surely shall have but little difficulty in understanding that, as in the inorganic world, so also in the world of organization, those all-pervading forces, which natural philosophers and chemists recognize, are constantly employed." (p. 14.)

To determine the operation of the imponderable agents upon the development of organized bodies, and especially upon that of plants, is the special object of Professor Draper's treatise. To say that he has been altogether successful, that his researches have exhausted the subject, or that some of them may not be hereafter set aside, would be to go far beyond his own estimate of the merits of his production. "Future discovery," he says (p. 2,) "in its progress, may show that, of the facts brought forward in this volume, many are misplaced, and many misapplied; these are incidents to which all philosophical works are liable. But if it should happen that anything contained herein shall aid in fastening the attention of men of science on the idea which it is designed to impart, the author will have received his reward, and the labours of ten years will not have been entirely thrown away." We may not altogether agree with our author, as to the degree of novelty which his conclusions possess; many of them, we believe, having been long familar to the minds of vegetable physiologists of the Old World. But we must do him the justice to state, that they are here set forth with a degree of precision which may be in vain looked for elsewhere, and that the mutual connexions of a large number of isolated facts in themselves familiar, are more fully elucidated than we have ever seen them before.

The first chapter contains a general account of the *action of the sunbeams in producing organized bodies;* sketching the influence of light on the processes of vegetation, from the development of the green flocks which are produced in spring water when exposed to the sun, or which cover damp surfaces, to the digestive functions of the leaves of more perfect plants, by which the carbon that is to be incorporated in their structure is obtained from the atmosphere. As we do not find any novelty in the contents of this chapter,—although we are bound to praise the lucid manner in which the subject is treated,—we shall pass on to the next, in which the author puts forth his views respecting the mechanical cause of the flow of the sap in plants, which he regards as fully explicable on the known principles of capillary attraction, and as analogous in its essential conditions to the circulation of the blood in animals. The physical phenomena of capillary attraction are treated in full in the appendix; which contains a valuable series of papers, on topics bearing on physiology, that have been at different times communicated by Dr. Draper to scientific publications. We must content ourselves with here concisely stating the *results* of his inquiries.

When one extremity of a small glass tube, open at both ends, is immersed in water, the water rises in the tube, to a height which bears a certain proportion to the bore of the tube. If it be immersed in mercury, however, the surface of the mercury within the tube is depressed; and the same result follows, if the interior of the tube be smeared with oil, and it be then immersed in water. "The physical law under which these elevations and depressions take place, is very simple and easy to be remembered. If a liquid can *wet* the surface of a solid, it will rise in a tube formed of that substance; but if a liquid *cannot wet* a solid, it will be depressed below its true level in a tube formed of that substance." Under no circumstance, however, will ordinary capillary action occasion a continuous flow from the top of a tube; for the liquid, having attained the highest possible elevation, remains there. But, if by evaporation (as in the case of an uncovered wick of a spirit-lamp,) or by chemical action or other processes (as in the combustion of oil in the wick of a lamp when lighted,) the superficial portions of the elevated liquid be removed from the extremity of the tube, a continuous flow up to that point will take place. These facts, which have long been recognized, are introduced, both by our author and ourselves, merely as preparatory to a higher order of phenomena,—those which are grouped under the name of endosmose. From his investigation of these phenomena, he deduces the following important law,—important not merely in itself, but from its bearing on physiological questions. "When two different liquids are brought in contact in a porous solid, which is wetted by both, but by them unequally, that one which has the greatest affinity for the solid, or which wets it most perfectly, will pass most rapidly through it, and may even drive the other entirely before it." This passage is not accomplished with an insignificant force. Direct experiment shows, that water will thus pass into alcohol, through a pervious membrane, with a force equal to the pressure of nearly two atmospheres. As a mechanical agent, capillary attraction, therefore, is fully able to overcome any of the resistances which it has to encounter in elevating sap to the tops of the loftiest trees, or in driving blood to the remotest parts of large animals.

In Prof. Draper's opinion, capillary attraction is itself but a result of electric action; and he brings forward many interesting facts in support of this view. We must pass by the subject, as being a question of physics, not of physiology; with the observation, however, that even though we may not be altogether prepared to assent to this position, we believe that those who have most attentively studied the molecular forces of bodies will be ready to admit the close relation between capillary attraction and chemical affinity; which is the point that Dr. Draper seeks to establish. Consequently the following modification of the principle just now stated, may be received as possessing a high degree of probability. "*If two liquids communicate with one another in a capillary tube, or in a porous or parenchymatous structure, and have for that tube or structure different chemical affinities, movement will ensue; that liquid which has the most energetic affinity will move with the greatest velocity, and may even drive the other fluid entirely before it.*" (p. 29.) We shall not stop to examine the application of this principle to the circulation of elaborated or *descending* sap in plants; because it does not appear to us to account very satisfactorily for its phenomena,—of which, indeed, we know very little. The *ascent* of the sap had been previously well explained on the physical principles applied to it by Dr. Draper. But we shall pass on to consider the explanation it gives of the movement of the blood in the capillaries of animals. We shall briefly follow Prof. Draper through his examination of this subject; and shall defer our own remarks upon it until its close.

The circulation in man (which, being most complex, may be regarded as including the phenomena of the circulation in general) may be considered under three heads,—the systemic, the pulmonary, and the portal. —The *arterial* blood, passing through the systemic capillaries, has a special affinity for the tissues through which it is transmitted; producing their oxydation, by the relinquishment of its own oxygen, which may be looked upon as its active principle. The *venous* blood, on the other hand, has little affinity for the structures with which it is in contact; the affinities of the arterial blood having been satisfied by the combustion of the tissues through which it has passed. According to the principle already stated, therefore, the arterial blood in the systemic capillaries will drive the venous blood before it, with a considerable amount of force. In the pulmonary circulation, on the other hand, the relative condition of the blood and the capillary channels is exactly the reverse. "We have here venous blood presenting itself on the air-cells, no longer presenting itself to carbonaceous or hydrogenous atoms, such as constitute the soft solids, but presenting itself to atmospheric air, or more truly to oxygen gas itself, which, being the more absorbable of the constituents of the air, is taken up and held in solution by the moist walls of the air-cells." This case, then, is precisely the converse of the former; since the stronger affinity is here between *venous* blood and the walls of the capillaries, so that the venous will drive the arterial blood before it. "Had we, therefore, known nothing of the circulation in the higher order of animals, but been instructed in the chemical relations of the blood to the soft tissues and atmospheric air, we could, upon physical principles, have predicted the existence of that circulation, and shown what its direction in different organs must be." In the portal circulation there are two forces at work, besides the impulse which the blood in the portal vein already possesses, by its transit through the capillaries of the chylopoietic viscera. The blood which arrives in the liver by the portal vein, undergoes a chemical change in that organ, the constituents of the bile being separated from it; and when the affinities which have been at work in producing this action have all been satisfied, the residue, which is inert *quoad* the liver, becomes the venous blood of the hepatic veins, and is consequently driven through the portal capillaries by the portal blood, whose affinities are yet to act. But besides this cause of movement, there is another, resulting from the operations which take place in the capillaries of the hepatic artery; these operations are upon the same footing with those of the systemic circulation in general, and will consequently give to the whole mass of the blood in the portal capillaries (into which the blood of the hepatic artery appears to be poured) a movement towards the vena cava.

Many physiologists (ourselves among the number) had arrived, before Dr. Draper took up the subject, at these conclusions in regard to the capillary circulation;—that it is in great part carried on by forces generated during its own continuance; and that the amount of these forces, determining the rate of movement of the blood, depended upon the activity of the actions going on between the blood and the tissues. Dr. Alison had further advanced the hypothesis, that a series of "vital attractions and repulsions" exists between the blood and the solids; the effect of which would be, to draw the particles of blood *towards* the solids, so long as they have not come into close relation with them, and afterwards to repel them. The idea of a series of attractions and repulsions, as the operating cause of the capillary circulation, was admitted by Dr. Carpenter; but he did not see reason to regard them as essentially distinct from those which operate in physics and chemistry. In Professor Draper's investigations, as it seems to us, a very striking confirmation of this last form of the hypothesis is to be found. He has shown that, admitting a difference of chemical relation between the arterial and venous blood, respectively, and the tissues through which the circulation is taking place, a movement of fluid must take place in a determinate direction; and that the direction of this movement is precisely that which we should expect, from our knowledge of the affinities in question, at least in the systemic and pulmonary circulation. The only error which we have to notice, is one which does not at all affect his *principle*, but which requires a modification in its application. Dr. Draper seems as if he regarded the affinity between the oxygen of the arterial blood, and the carbonaceous and hydrogenous atoms which are ready to be burned out of the tissues, as the *only* one involved in the production of the movement. For anything that he says to the contrary, we might suppose that arterial blood is *merely* a carrier of oxygen to the tissues; and the venous blood a return carrier of carbonic acid and water. We believe that this simple kind of action is what *does* obtain (of course under a reversed form) in the *pulmonary* circulation; that is, that the discharge of carbonic acid and the imbibition of oxygen are, on the principles enunciated by Dr. Draper, the essential causes of the motion of the blood through the pulmonary capillaries, in a direction from the venous towards the arterial side of the circulation. But the case is very different with respect to the actions to which the blood is subservient in the *systemic* capillaries; for the deoxygenation of the arterial blood, and the combustion-process in the tissues, constitute only one of a very complex series of changes, to which the blood is there subservient.

We think, therefore, that *all* these changes ought to be taken into the account; and that, when this is done, the theory will be as perfect as, in the present state of our knowledge, it can be well made.

Here we must lay down, for the present, this very interesting volume. A large portion of the remainder of it is occupied by an account of Dr. Draper's investigations into the mode in which *light* acts upon vegetables; and the varying operations, in this respect, of different parts of the solar spectrum. Inquiries upon this subject are now being actively prosecuted by experimenters in different parts of the world; but their results are not yet sufficiently accordant, to enable us to speak positively on the subject. We may hereafter take an opportunity of adverting to the subject; and shall for the present take our leave of Prof. Draper; expressing our thanks to him for the important contribution which he has made to physiological science, and our hope that he will continue to pursue the same line of investigation with increasing success.

---

